# Gene signature associated with neuro‐endocrine activity predicting prognosis of pancreatic carcinoma

**DOI:** 10.1002/mgg3.729

**Published:** 2019-05-17

**Authors:** Ke Chen, Yiping He, Yuan Liu, Xiujiang Yang

**Affiliations:** ^1^ Department of Endoscopy Fudan University Shanghai Cancer Center Shanghai China; ^2^ Department of Oncology Shanghai Medical College, Fudan University Shanghai China

**Keywords:** carcinoma, neuro‐endocrine, pancreas, prognosis, TCGA

## Abstract

**Background:**

Genomic analysis is the promising tool to clear understanding of the tumorigenesis and guide molecular classification for pancreatic cancer. Our purpose was to develop a critical predictive model for prognosis in pancreatic carcinoma, based on the genomic data.

**Methods:**

The online The Cancer Genome Atlas (TCGA) and International Cancer Genome Consortium (ICGC) datasets were queried as training and validation cohorts for comprehensive bioinformatic analysis. We applied Lasso and multivariate Cox regression to shrink genes and construct predictive model.

**Results:**

A four genes model (DNAH10: HR = 0.71, 95% CI = 0.57–0.88, HSBP1L1: HR = 1.51, 95% CI = 1.18–1.92, KIAA0513: HR = 0.69, 95% CI = 0.50–0.96, and MRPL3: HR = 3.73, 95% CI = 2.03–6.86), was proposed and validated. The C‐index was 0.73 (95% CI: 0.7–0.77). Patients in high‐risk and low‐risk group, stratified by model, suffered significantly different overall survival time (15.1 vs. 49.3 months, *p* < 0.0001 in TCGA; 423 vs. 618 days, *p* = 0.038 in ICGC). Taken clinical parameters into consideration, the risk‐score was independent marker in clinical subpopulation. To explore the molecular mechanisms, 579 differential expression genes (DEG) in two groups were identified by *edgeR*. Functional enrichment of DEG indicated neuro‐endocrine activity was the potential mechanism for the discrepant prognosis.

**Conclusion:**

A specific four genes signature with the ability to predicted survival of pancreatic carcinoma was generated, which may indicate the connection between neuro‐endocrine activity and patients’ prognosis.

## 1 INTRODUCTION

Pancreatic cancer is one of the most lethal malignancy. According to the cancer statistics in 2017, estimated death in USA for pancreatic cancer were 22,300 cases and 20,790 cases, both accounted for the 7% of all cases in men and women (Siegel, Miller, & Jemal, 2017). Most patients were diagnosed with advanced stage and unresectable status (Karakas, Lacin, & Yalcin, 2018). Besides, response rate for first‐line chemotherapy regimens was quite dissatisfactory. What was worse, no novel‐targeted therapies for pancreatic cancer were proved to be effective, which made the clear understanding of tumorigenesis urgent for the drug discovery.

As to the foremost organ with exocrine and endocrine functions, the molecular features in pancreatic carcinogenesis were unique and mostly unknown (Chandra & Liddle, 2013; Venkatesh & Monje, 2017). Research efforts have been focused on the genomic landscapes of pancreatic cancer, based on the RNA micro‐array or sequence technology (Notta, Hahn, & Real, 2017). The numerous genomic features make future managements more specific and individualized. As a consequence, deep and comprehensive interpretation of those genomic data owes great value to uncover the propagable predictors in the risk estimation of carcinogenesis, therapeutic response, and prognosis. Here, we established a four genes signature for pancreatic cancer prognosis based on The Cancer Genome Atlas (TCGA) database, and validated in further International Cancer Genome Consortium (ICGC) database. This gene signature indicated the critical value of neuro‐endocrine activity in the pancreatic cancer.

## 2 MATERIAL AND METHODS

### 2.1 Ethical compliance

This is a secondary analysis based on the open online‐databases. As reported in the original database, all procedures performed in studies involving human participants were in accordance with the ethical standards of the institutional and/or national research committee. Informed consent was obtained from all individual participants included in the original studies.

### 2.2 Data sources and processing

Genomic data of RNA sequence for pancreatic carcinoma were queried from TCGA database and ICGC database. The included criteria for the analysis were: (a) pathological type was pancreatic carcinoma; (b) overall survival (OS) data were available; (c) raw count or normalized gene expression data were available. After the screening, project PAAD from TCGA and PACA‐AU from ICGC were obtained, with the available clinical data. All the data were queried and extracted on January 1, 2018. We predefined the PAAD as training set, PACA‐AU as validation set. To control the heterogeneity, log2 transformed normalized read counts extracted from “rsem.genes.normalized_results” files in TCGA and raw Z‐scores for ICGC were adopted for normalization. Finally, we totally enrolled 178 and 84 pancreatic carcinoma cases for TCGA and ICGC, respectively.

### 2.3 Survival signature development

**Figure 1:**
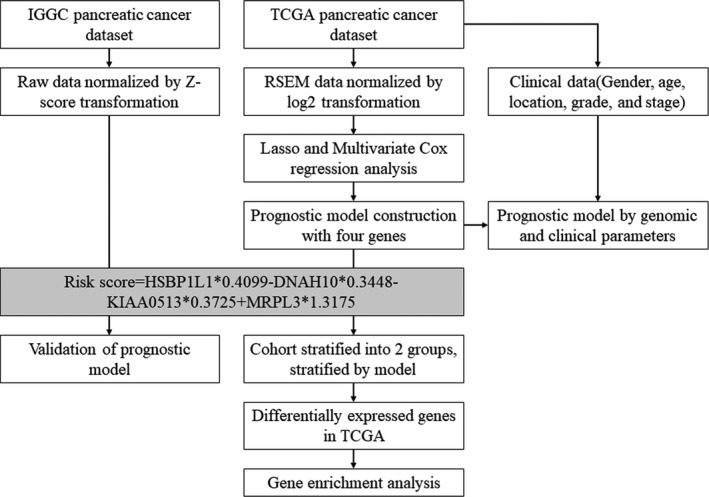
Flow‐chart of the bioinformatic analysis

To select the predictive genes for patients’ survival, L1 penalized Lasso regression was initially performed, which was suitable for high‐dimension genomic dataset (Friedman, Hastie, & Tibshirani, 2010). With the variable selection and shrinkage, interpretable prediction genes were further delivered for multivariate Cox regression to construct the survival model. We calculated the risk‐score for each pancreatic carcinoma patients based on the individual expression levels of selected genes, where $riskscore=\sum_{i=1}^{n}\beta_{i}\times exp\left(G_{i}\right)$ (Shukla et al., 2017). In the equation, n genes were enrolled as variables, exp(*G_i_*) represented the normalized expression of gene *i*, while *β_i_* represented the coefficient for gene *i*. We set the median of risk‐score as the cutoff value, and patients were stratified as high‐risk group with risk‐score ≥ median and low‐risk group with risk‐score < median. Due to the censored data in survival analysis, we selected the C‐index in the model assessment. For survival analysis, we applied Kaplan–Meier method to calculate OS time in different risk group. The log‐rank test was performed to check the statistical significance. The unpaired *t* test was applied to assess whether a selected prediction gene was differentially expressed between two risk groups. Statistical significance was determined using *p* < 0.05. All the analyses were performed in R software. Glmnet (Friedman et al., 2010), survival (Therneau & Grambsch, 2000), and survminer/ggplot2 (Hadley, 2016) packages implemented in R software were called for lasso regression, cox regression, survival analysis, and data visualization, respectively. Forest plots were drawn to demonstrate the hazard ratio (HR) of selected prediction genes. Expression of the four genes signature and clinical profile were visualized by Complex‐Heatmap. The flow chart was shown in Figure 1.

### 2.4 Differential gene expression and functional enrichment analysis

To investigate the genomic profile between high and low‐risk group, we further performed the differential expression genes (DEG) analysis by edgeR Package in TCGA cohorts.(Robinson, McCarthy, & Smyth, 2010) Fold change and false discovery rate (FDR) were set as 2 and 0.05. Volcano plots were drawn to visualize the DEG. DEG were further delivered for gene ontology and KEGG pathway enrichment, which were performed by over‐representation analysis with Fisher's exact test, and Benjamini–Hochberg multiple test to correct occurrence of false positive. Strict cutoff of *p* < 0.01 and FDR <0.05 was set. The statistics and data visualization were performed by ClusterProfiler Package in R software (Yu, Wang, Han, & He, 2012).

### 2.5 Clinical phenotype analysis

Predictive value of the four genes signature in different clinical profiles was also investigated. We enrolled the risk‐score, gender, age, tumor location, grade, and stage as confounders to calculated the multivariate Cox regression. Besides, survival status of high‐risk and low‐risk groups in different clinical subpopulations were checked.

### 2.6 Statistics

All analyses were set at two‐sided *p *< 0.05 as the threshold for statistical significance. The data were expressed as mean ± standard deviation.

## 3 RESULTS

### 3.1 Clinical characteristics of the included cohorts

With the inclusion criteria, project PAAD from TCGA and PACA‐AU from ICGC were obtained (Table 1). In TCGA cohort, 178 patients were analysis, with an average age of 64.6 (±10.9) years. Most patients were males (*n* = 98) and presented with tumors in pancreatic head (138). The histological grade included 30 for G1, 96 for G2, 47 for G3, two for G4, and three for Gx. According to American Joint Committee on Cancer staging system, T stages included T1 (*n* = 6), T2 (*n* = 24), T3 (*n* = 142), T4 (*n* = 3), and Tx (*n* = 3), N stages included N0 (*n* = 49), N1 (*n* = 123), and Nx (*n* = 6), M stages included M0 (*n* = 78), M1 (*n* = 5), and Mx (*n* = 95). The average OS time was 18.8 (±15.6) months. In the ICGC cohort, 84 patients were retrieved, including 38 females and 46 males, with an average age of 66.2 (±11.4) years. Tumors located in pancreatic head were presented in 66 cases, and the average OS time was 542.3 (±374.9) days.

**Table 1:** Clinical parameters of the TCGA and ICGC cohorts

	TCGA training‐cohort (*n* = 178)	ICGC validation‐cohort (*n* = 84)
Age (mean ± *SD*)	64.6 (±10.9)	66.2 ± 11.4
Gender (male/female)	98/80	46/38
Location		
Head/body/tail/other	138/14/15/11	66/4/14/0
AJCC T stage		
T1/T2/T3/T4/Tx	6/24/142/3/3	NA
AJCC N stage		
N0/N1/Nx	49/123/6	NA
AJCC M stage		
M0/M1/Mx	78/5/95	NA
Over‐all survival time (mean ± *SD*)	18.8 ± 15.6 months	542.3 ± 374.9 days
Histological type		
Ductal adenocarcinoma	148	70
Other	30	14
Histological grade		
G1/G2/G3/G4/Gx	30/96/47/2/3	31/15/20/18/0

Abbreviations: ICGC, International Cancer Genome Consortium; TCGA, The Cancer Genome Atlas; NA, not available.

### 3.2 Generation of a four genes prognostic signature in TCGA cohorts

**Figure 2:**
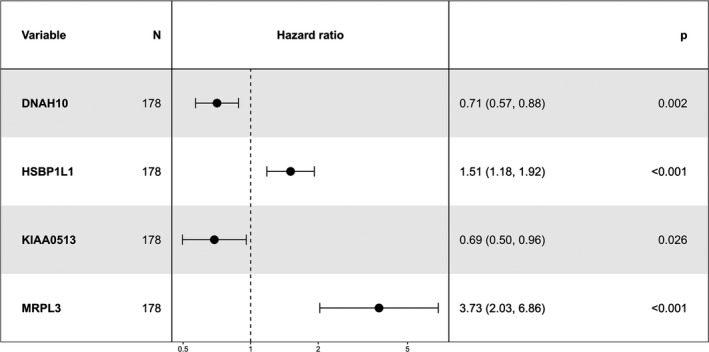
Forest plot of the four genes signature in the predictive model for overall survival

**Figure 3:**
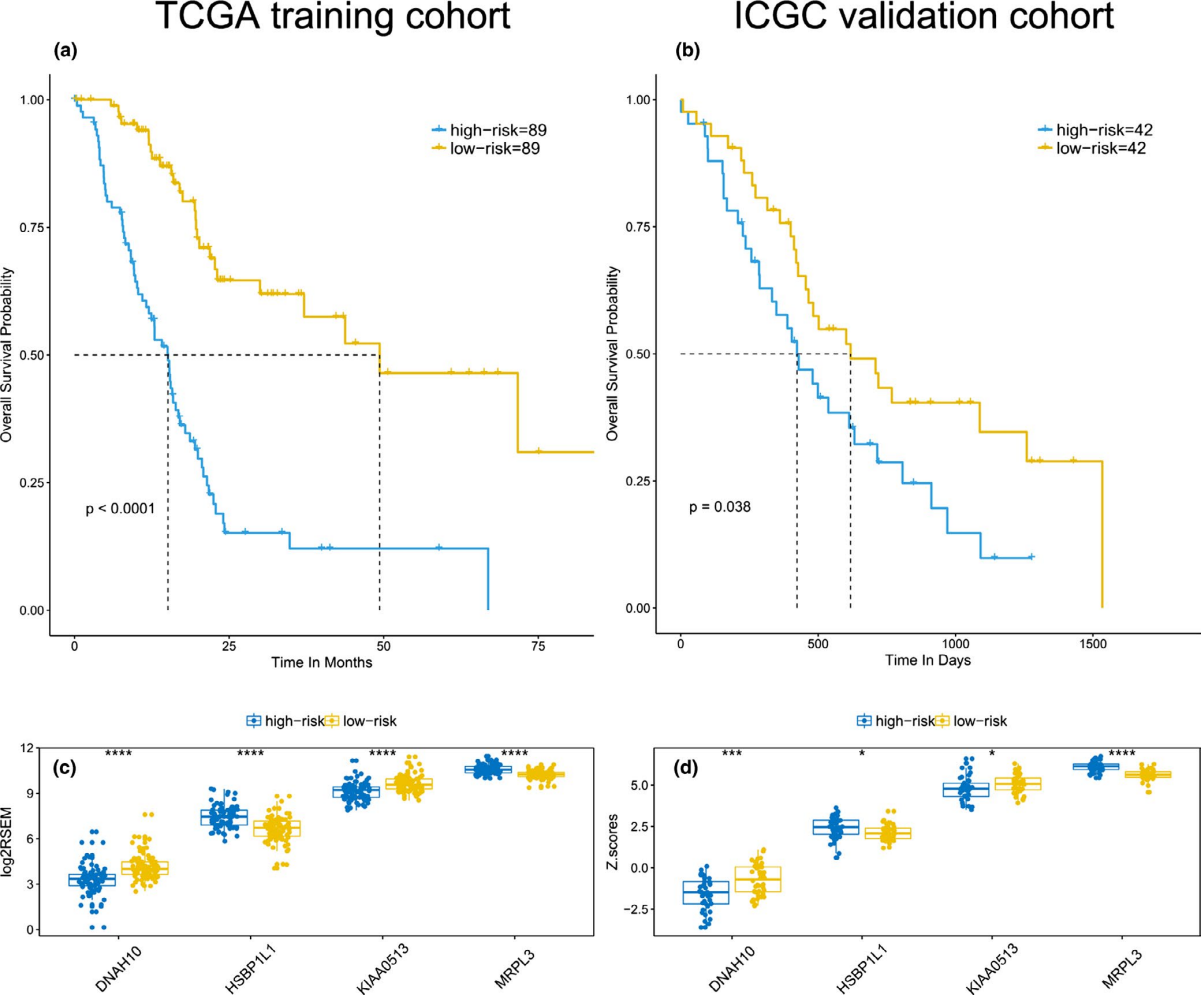
Performance and expression of the four genes signature in TCGA training and ICGC validation cohorts. The Kaplan–Meier plot indicated high‐risk group had significantly worse prognosis than low‐risk group in TCGA cohort (a) and ICGC cohort (b). Expression level of the four predictive genes were significantly different in two groups of TCGA cohort (c) and ICGC cohort (d). One star indicated *p* < 0.05, three stars indicated *p* < 0.001, four stars indicated *p* < 0.0001. ICGC, International Cancer Genome Consortium; TCGA, The Cancer Genome Atlas

**Figure 4:**
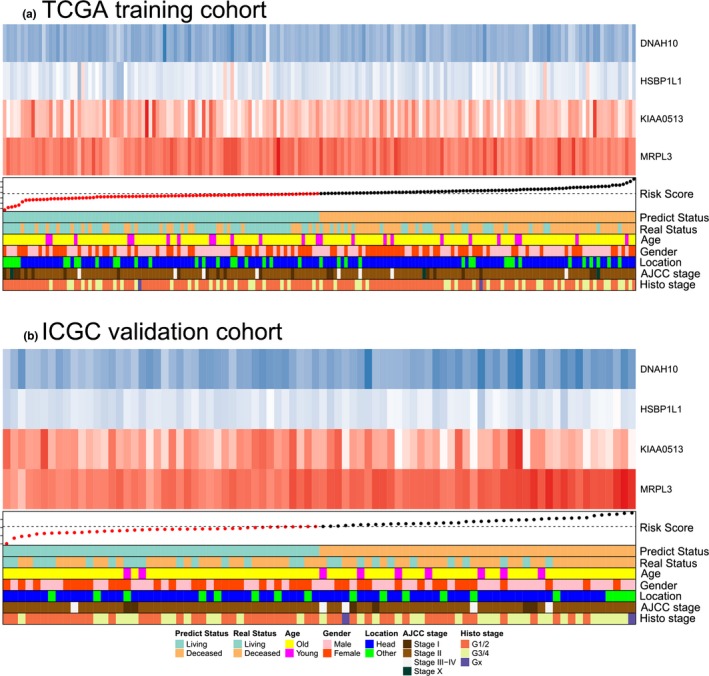
Heatmap demonstrated the expression of the four predictive genes with distribution of risk‐score, age, gender, location, grade, and stage in TCGA cohort (a) and ICGC cohort (b). The blue box in the heatmap indicated the low expression, and red box indicated the high expression. Dashed line in the annotation indicated the median value of risk score. ICGC, International Cancer Genome Consortium; TCGA, The Cancer Genome Atlas

We set the TCGA cohort as the training set, and mapped genes in the expression matrix. Lasso regression analysis was initially applied to shrink the high‐dimension genomic data. Totally, 22 genes were selected with non‐zero regression coefficients at the value of λ with optimal cross‐validated likelihood, including ARNT2, ARNTL2, B3GNT8, C6orf122, CASKIN2, CDK6, CYP27A1, DNAH10, HSBP1L1, INSIG2, KIAA0195, KIAA0513, LQK1, MRPL3, PI4KB, PPP2R3A, RARRES3, RPAP2, SEC16A, SEC61A2, THSD1P1, and TMEM104. According to the annotation in Pubmed, C6orf122 was non‐coding RNA and removed in further analysis. Next, the 21 genes were delivered to multivariate Cox regression analysis in the same cohort. The result indicated four genes, DNAH10, HSBP1L1, KIAA0513, and MRPL3, were the independent predictors for prognosis. The detailed information was shown in Table S1. Subsequently, a four genes model was formulated, based on the risk‐score = HSBP1L1 × 0.4099 − DNAH10 × 0.3448‐KIAA0513 × 0.3725 + MRPL3 × 1.3175. The C‐index was 0.734 (95% CI: 0.7–0.768) for the four genes model, which indicated the superiority of the model for predicting OS in pancreatic carcinoma. The detailed HR, coefficient, and *p*‐value were presented in Table 2 and Figure 2. According to the risk‐score, TCGA cohort was stratified into high‐risk group with risk‐score ≥ median and low‐risk group with risk‐score < median. The Kaplan–Meier plot indicated high‐risk group had significantly worse prognosis than low‐risk group (*p* < 0.0001), with median OS time were 15.1 and 49.3 months, respectively (Figure 3a). Expression level of the four predictive genes were significantly different in two groups (Figure 3c). The positive prognostic factors, HSBP1L1 and MRPL3, were both high‐expression in high‐risk group, while the negative factors, DNAH10 and KIAA0513 were low‐expression in high‐risk group. Value of the four genes signature in different clinical profile was analyzed. Stratification by patients’ age, gender, location, tumors’ grade, and stage, the risk‐score independently predicted prognosis in different subpopulation (Figure S1). In the meantime, we also inputted the clinical parameters with risk‐score in Cox regression analysis, and got the integrated‐model, in which risk‐score and histological grade were the independent predictors for prognosis. The C‐index was 0.696 (95% CI: 0.663–0.729) for the integrated‐model. The C‐index was 0.745 (95% CI: 0.711–0.779), when the four selected genes (each treat as continuous variable) and clinical variables as the covariates, which was similar with the four‐gene‐model. Furthermore, when only histological grade was considered without the risk score, the C‐index was 0.549 (95% CI: 0.522–0.576). Hence, based on those results, the risk score was the major contribution to the prognosis prediction. The heatmap demonstrated the expression of the four predictive genes with distribution of risk‐score, age, gender, location, grade, and stage in TCGA cohort (Figure 4a). Taken all together, the four genes signature acted very well to predict the prognosis for pancreatic carcinoma.

**Table 2:** The multivariate cox regression analysis for OS

Parameters	Coefficient	*p* value	HR (95% CI)
Four genes model (only four candidate genes were shown)			
DNAH10	−0.3448	**0.0021**	0.71 (0.57–0.88)
HSBP1L1	0.4099	**<0.0001**	1.51 (1.18–1.92)
KIAA0513	−0.3725	**0.0258**	0.69 (0.50–0.96)
MRPL3	1.3175	**<0.0001**	3.73 (2.03–6.86)
Integrated‐model (all the included factors were shown)			
Risk‐score (low‐risk vs. high‐risk)	−1.3258	**<0.0001**	0.27 (0.16–0.44)
Age (≦50y vs. >50y)	0.1847	0.5809	1.20 (0.62–2.32)
Location (other vs. head)	−0.5968	0.0686	0.55 (0.29–1.83)
Gender (male vs. female)	−0.1167	0.5998	0.89 (0.58–1.38)
AJCC T stage (T3/4 vs. T1/2)	−0.1078	0.7670	0.90 (0.44–1.83)
AJCC M stage (M1/x vs. M0)	−0.1280	0.5594	0.88 (0.57–1.35)
AJCC N stage (N1/x vs. N0)	0.4735	0.1018	1.61 (0.91–2.83)
Histological grade (G3/4 vs. G1/2)	0.5324	**0.0248**	**1.70 (1.07–2.71)**

Abbreviations: HR, hazard ratio; OS, overall survival.

### 3.3 Validation of the four genes prognostic signature in ICGC cohort

To check the external validity and general applicability, we applied our four genes model in RNA sequence data of ICGC. The C‐index was 0.637 (95% CI: 0.594–0.680) for the ICGA cohort. According to the four genes model, low‐risk group survived longer than high‐risk group (*p* = 0.038), with median OS time were 618 and 423 days, respectively (Figure 3b). Be similar to TCGA cohorts, both HSBP1L1 and MRPL3 were highly expressed, DNAH10 and KIAA0513 were lowly expressed in high‐risk group (Figure 3d). The detailed HR and *p*‐value were presented in Figure S2, in which low‐expressed DNAH10 and KIAA0513 were proved to be robust for prognosis prediction. Thus, the four genes signature was potential prognostic marker for pancreatic carcinoma, based on RNA sequence data.

### 3.4 Differential gene expression and functional enrichment analysis

**Figure 5:**
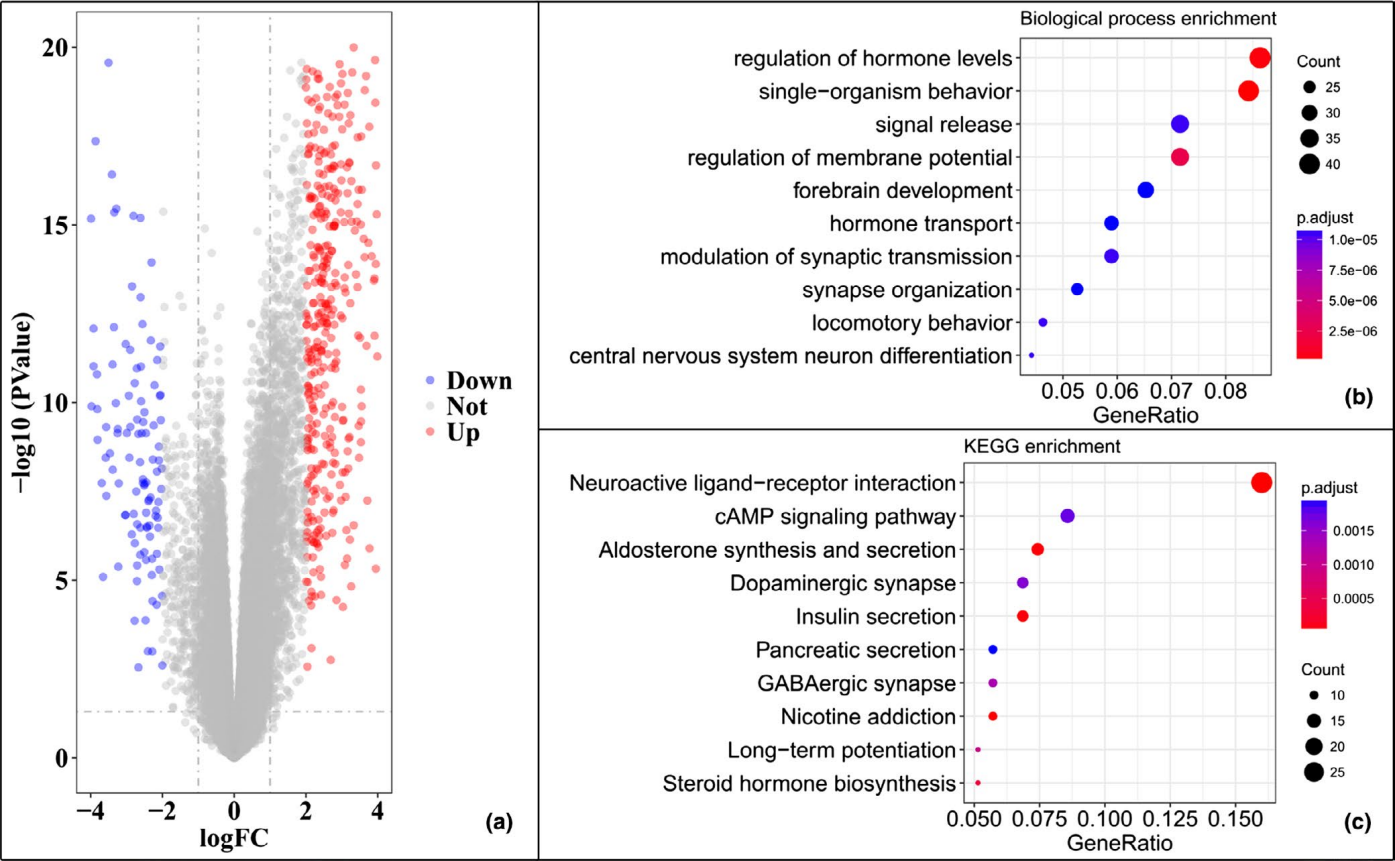
Differential gene expression and gene functional enrichment analysis. The volcano plot shown the 579 DEG identified in TCGA cohort (a). The functional enrichment included biological process (b) and KEGG pathway (c). The top 10 items for two sets were shown. DEG, differential expression genes; TCGA, The Cancer Genome Atlas

To reveal the important molecular events, accounted for the prognosis, DEG were calculated in TCGA cohort. As shown in the volcano plot (Figure 5a), a list of 579 genes were identified, of which 454 genes were up‐regulated, and 125 genes were down‐regulated. Besides, the relevant biological process and signal pathway were mapped by over‐representation analysis. Ultimately, the neuro‐endocrine signal transition including regulation of hormone, synaptic, signal release, and membrane potential was the most enrichment biological process (Figure 5b). Corresponding, the regulated pathways were majorly focused on the neurotransmitter activities (Figure 5c).

## 4 DISCUSSION

The genomic background drives the biological phenotypes, which also may be retro‐regulated by the external environments. Even for cancers, the unheeded genomic profiles gradually exerted great predictive values for clinical decision‐making, such as early detection of precancerous, optimization of therapeutic strategies, and disease prognosis. In this work, we proposed a risk‐score formula by four genes signature for pancreatic carcinoma, which was proved to be feasible and creditable for prognostic prediction.

To establish the predictive model, we initially applied Lasso algorithm to shrink the candidate genes in the whole genomic set. Subsequently, multivariate cox regression was called to construct the formula. Then a four genes model was constructed and suggested. This mining regime was reported quite suitable for predictors filtering in genomic data. Furthermore, this four genes model was also validated in external RNA sequence cohort. Living or deceased outcomings were very consistent with the risk group, stratified by gene signature, both in training and validation cohorts. When considered this signature in the clinical set, the predictive value was also robust in different subpopulation. Studies indicated early and late onset pancreatic cancer suffered different genomic alteration and clinicopathologic features (McWilliams et al., 2016; Ohmoto et al., 2016). Survival analysis in the TCGA cohort was not available to support this point, but our risk model was both feasible in patients with diagnostic age larger or <50 (Figure S1). Similarly, tumor histological grade and AJCC stage did not influence the application of our risk model. These results indicated our risk model was an independent factor for prognosis.

In this four genes signature, two genes (HSBP1L1 and MRPL3) were positively related to prognosis, and the other two (DNAH10 and KIAA0513) were negative factors, when set the OS as the primary outcome. For the four genes, evidences presented in the current study provided, to our knowledge, the first link with pancreatic cancer prognosis. HSBP1L1 was a 72 amino‐acid protein and showed 41.2% identity with HSBP1 (heat shock transcription factor binding protein 1). But no reports about the exact functions for HSBP1L1 in biology. Presumably, HSBP1L1 might suppress the heat shock factor transcription under stress. MRPL3 encoded a 39s subunit protein that belonged to the large mitochondrial ribosomes family. Previous report indicated MRPL3 was the candidate susceptibility genes for common familial colorectal cancer and RNA metabolism‐related genes in non‐small cell lung cancer (Gylfe et al., 2013; Valles et al., 2012). DNAH10 was an inner arm dynein heavy chain, and reported to involve with pathogenesis of human insulin resistance (Lotta et al., 2017). KIAA0513 was the most investigated gene and mainly expressed in normal brain tissues (Lauriat et al., 2006). Functional analysis indicated KIAA0513 participated into the neuroplasticity, apoptosis, and cytoskeletal regulation, and it was therefore reasonable that the gene seemed to represent the neuro‐endocrine activity in pancreatic cancer.

Nerve is the common feature in the niche of pancreatic cell, not only for the islet cells, but also for the duct epithelium and acinar cells. Stimulated by the nervous impulse, pancreatic cells make series of biological responses, in the action of neurotransmitters. Numerous evidences indicated the role of neuronal activity in none‐nervous organs for carcinogenesis, especially for pancreas, prostate, and gastrointestinal system (Magnon et al., 2013; Saloman et al., 2016; Venkatesh & Monje, 2017). In vivo model conducted by Saloman et al. (2016) forcefully proved pancreatic sensory neurons supported the initiation and progression of pancreatic carcinoma. By the gene enrichment analysis, we found that differential genes in high‐risk and low‐risk groups were majorly focused on the neuro‐endocrine activity, such as the neuroactive ligand–receptor interaction (KEGG: hsa04080). Next, biosynthesis and secretion of neurotransmitters were also frequently hit, including aldosterone, dopamine, insulin, gamma‐aminobutyric acid (GABA), and nicotine. To be encouragingly, most of those factors were reported previously to correlate with pancreatic cancer. Genomic data of Jandaghi et al. (2016) found that dopamine receptor D2 was significantly upregulated both in RNA and protein level in pancreatic cancer and inhibitors suppressed tumor growth in mice. Clinical observation indicated fasting insulin was causally associated with an increased risk of pancreatic cancer (Carreras‐Torres et al., 2017). Moreover, psychological stress also might worse the clinical prognosis in pancreatic cancer, via the neurotransmitter GABA (Schuller, Al‐Wadei, Ullah, & Plummer, 2012). Binding of nicotine to the receptors in pancreatic cell stimulated the secretion of autocrine catecholamine and promoted cell proliferation (Al‐Wadei, Al‐Wadei, & Schuller, 2012). All these reported evidences and our current analysis indicated the great value of the neuro‐endocrine activity in pancreatic cancer progression.

There were several shortcomings to our study. First, due to difference in data processing, external applicability of the four genes model in the microarray matrix was not warranted, which was not validated in our study. Second, pathogenesis and molecular events might be different in histological subtypes of pancreatic carcinoma. Totally, 83.1% and 83.3% cases were ductal adenocarcinomas in TCGA and ICGC cohorts, which were the most common subtypes. Hence, the unavoidable heterogeneity might weak the interpretation of the four genes model for a specific subtype of pancreatic carcinoma. Third, it was hard to validate the integrated‐model in ICGC cohort, due to the unavailable clinic profiles. But the integrated‐model was not superior to the four genes model in TCGA cohort. It indicated the combination with clinical profile might not be suitable for genetic profile, but not ruled out the possibility of a better result based on the ICGC cohort, if clinical profile was available. Finally, all the four genes had not been previously reported in pancreatic cancer. Analysis based on computational software needed further in‐home validation beside the bench.

In conclusion, on the basis of TCGA sequence data, we proposed a four genes model (DNAH10, HSBP1L1, KIAA0513, and MRPL3), which facilitated the discernment of high‐risk patients for worse OS in pancreatic carcinoma. This model also demonstrated fine performance in external ICGC validation cohort. Taken clinical parameters into consideration, the risk‐score was independent marker in each clinical subpopulation. Furthermore, DEG and functional enrichment indicated the neuro‐endocrine activity was the potential mechanisms for the prognosis prediction. Future large cohorts and basic experiments were warranted to verify this four genes model and regulation of the four genes in neuro‐endocrine pathway.

## CONFLICT OF INTEREST

None declared.
