# Supplementary figures and images for: Gene signature associated with neuro‐endocrine activity predicting prognosis of pancreatic carcinoma
Source: Mol Genet Genomic Med. 2019 May 17;7(7):e00729. doi: 10.1002/mgg3.729 (PMC6625361; doi:10.1002/mgg3.729)

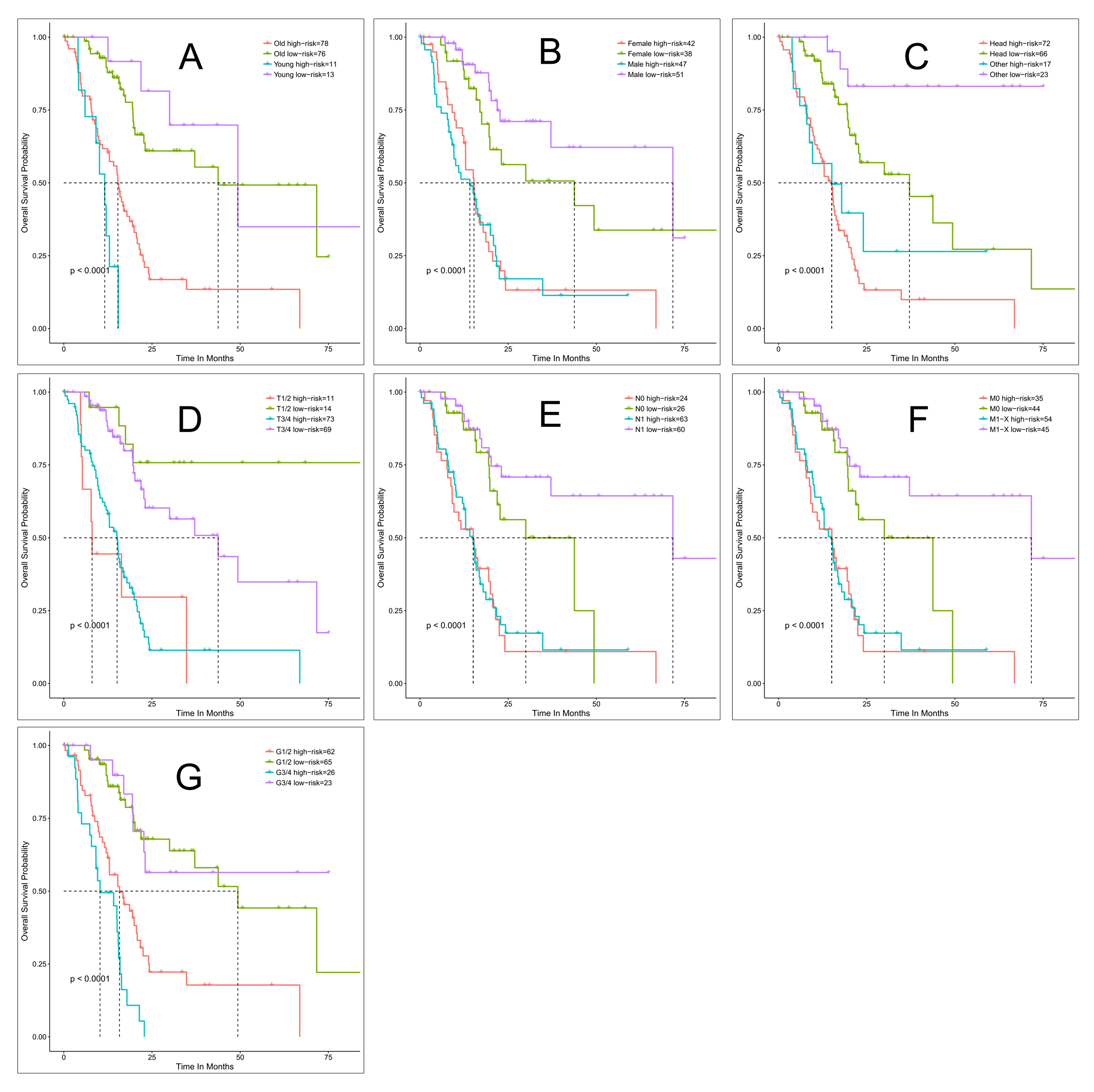

Supplement: Supplementary file 1 [file MGG3-7-e00729-s001.tif]
